# 1170. Do Rotavirus Strains Affect Vaccine Effectiveness? A Systematic Review And Meta-analysis

**DOI:** 10.1093/ofid/ofab466.1363

**Published:** 2021-12-04

**Authors:** Jordan Cates, Avnika Amin, Jacqueline Tate, Ben Lopman, Umesh D Parashar

**Affiliations:** 1 Division of Viral Diseases, National Center for Immunization and Respiratory Diseases, Centers for Disease Control and Prevention, Atlanta, Georgia; 2 Department of Epidemiology, Emory University Rollins School of Public Health, Atlanta, Georgia; 3 Division of Viral Diseases, Centers for Disease Control and Prevention, Atlanta, GA; 4 Rollins School of Public Health, Emory University, Atlanta, GA

## Abstract

**Background:**

Rotavirus causes 215,000 deaths from severe childhood diarrhea annually. Two rotavirus vaccines – a monovalent vaccine containing a single rotavirus strain (RV1) and a pentavalent vaccine containing 5 rotavirus strains (RV5) – are used in routine immunization programs of nearly 100 countries. Concerns exist that rotavirus vaccines may be less effective against rotavirus strains not contained in the vaccines which could subsequently cause selective pressure and strain replacement. We estimated the vaccine effectiveness (VE) of RV1 and RV5 against vaccine (homotypic) and non-vaccine (partially and fully heterotypic) strains.

**Methods:**

After conducting a systematic review, we meta-analyzed 31 case-control studies (N=27,293) conducted between 2006 and 2020 using a random-effect regression model.

**Results:**

In high-income countries, RV1 VE was 10% lower against partially heterotypic (p-value=0.04) and fully heterotypic (p-value=0.10) compared to homotypic strains (homotypic VE: 90% [95% CI: 82, 94]; partially heterotypic VE: 79% [95% CI: 71, 85]; fully heterotypic VE: 80% [95% CI: 65, 88]; Figure 1). In middle-income countries, RV1 VE was 14 to 16% lower against partially heterotypic (p-value=0.06) and fully heterotypic (p-value=0.04) compared to homotypic strains (homotypic VE: 81% [95% CI: 69, 88]; partially heterotypic VE: 67% [95% CI: 54, 76]; fully heterotypic VE: 65% [95% CI: 52, 75]; Figure 1). Strain-specific RV5 VE differences were less pronounced (Figure 2). Limited data were available from low-income countries.

Figure 1. Vaccine effectiveness by country income level and strain type, for RV1.

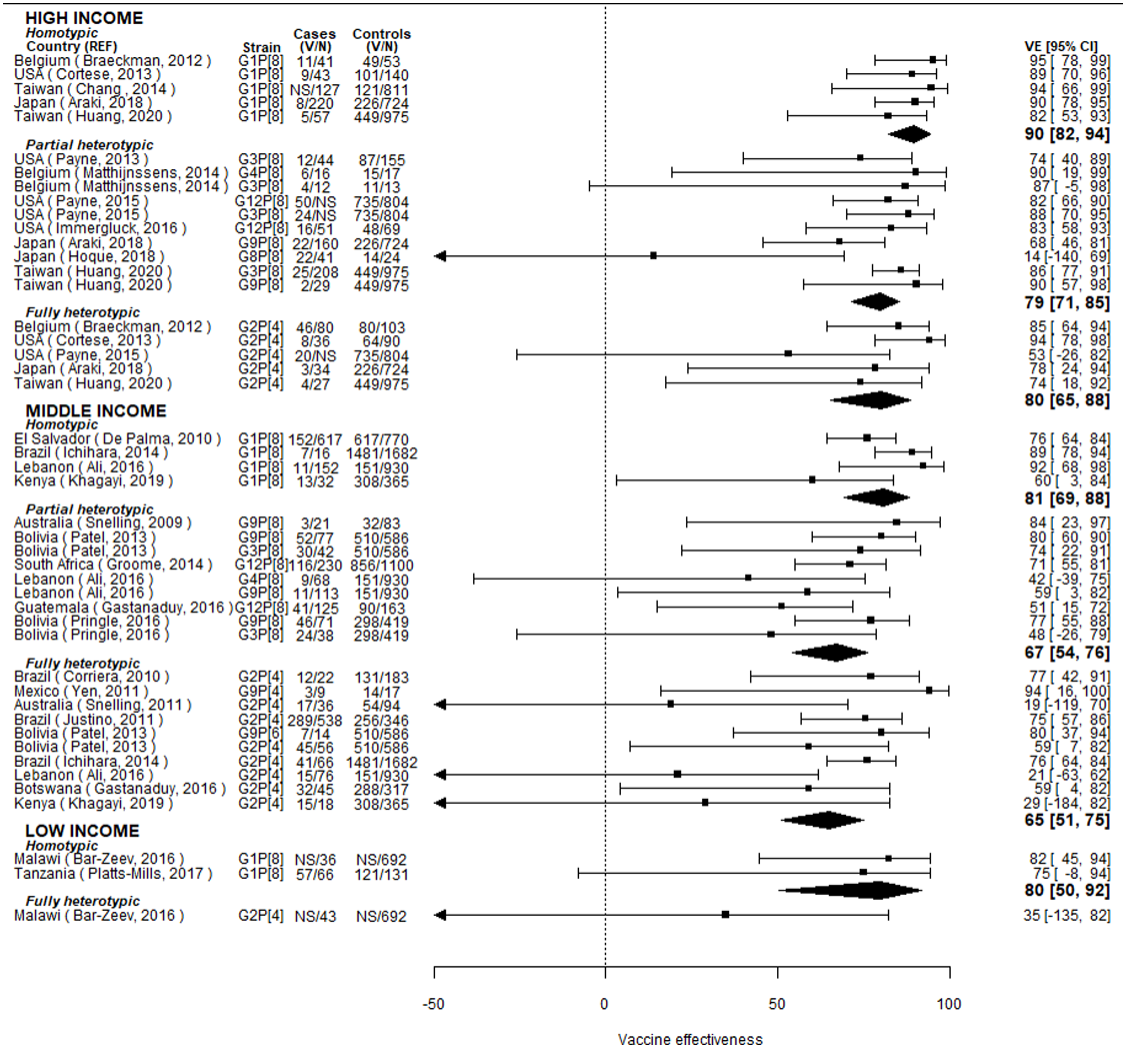

Figure 2. Vaccine effectiveness by country income level and strain type, for RV5.

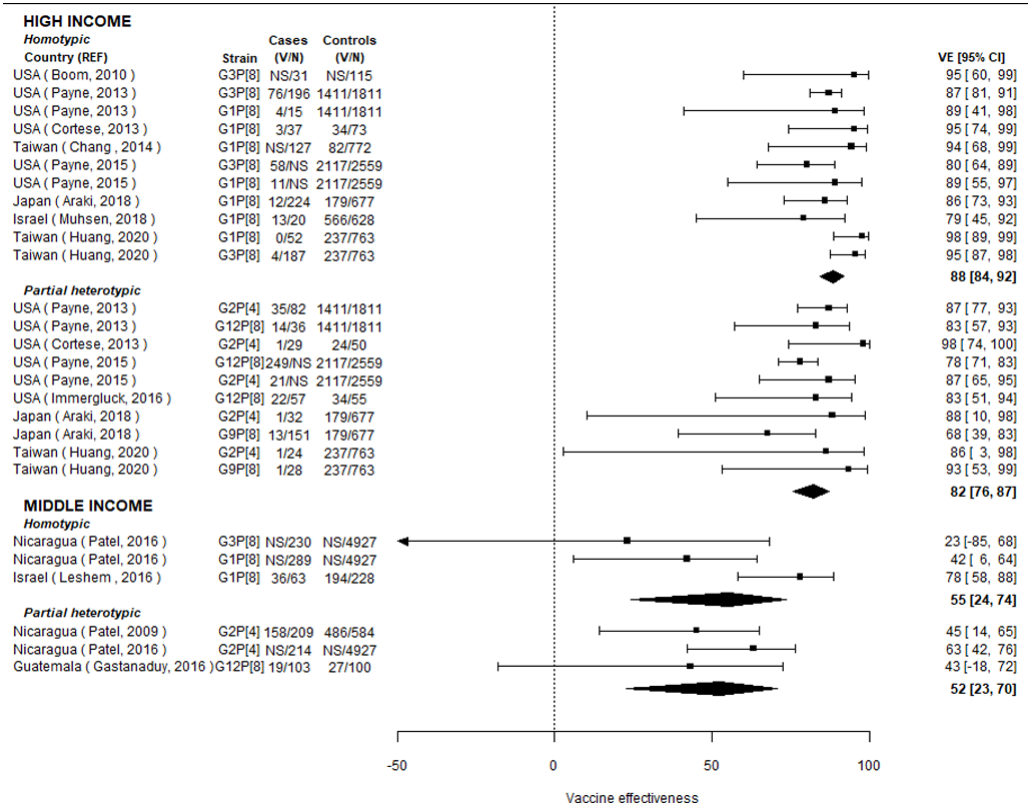

**Conclusion:**

Vaccine effectiveness of RV1 and RV5 was somewhat lower VE against non-vaccine than vaccine strains. Ongoing surveillance is crucial to continue long-term monitoring for strain replacement, particularly in low-income settings where data are limited.

**Disclosures:**

**All Authors**: No reported disclosures

